# Editorial: Fat Metabolism and Deposition in Poultry: Physiology, Genetics, Nutrition and Interdisciplinary Research, Volume I

**DOI:** 10.3389/fphys.2022.937081

**Published:** 2022-05-26

**Authors:** Sami Dridi, Craig W. Maynard, Jie Wen, Elizabeth R. Gilbert

**Affiliations:** ^1^ Center of Excellence for Poultry Science, University of Arkansas, Fayetteville, AR, United States; ^2^ Institute of Animal Sciences, Chinese Academy of Agricultural Sciences, Beijing, China; ^3^ Department of Animal and Poultry Sciences, Virginia Tech, Blacksburg, VA, United States

**Keywords:** fat metabolism, chickens, avian, nutrition, omics

The intensive genetic selection of broiler (meat-type) chickens over the past 80 years has focused narrowly on economically important traits, namely growth rate, feed efficiency, and breast yield (Griffin and Goddard, 1994). Although this has led to spectacular progress in term of productivity and profitability which helped in supporting the livelihoods and food security of billions of people worldwide, there are several unexpected and undesirable changes, including hyperphagia (Denbow, 1994), metabolic disorders such as leg problems (Julian, 1998), breast muscle myopathies (Velleman, 2015), cardiovascular diseases (Olkowski, 2007), and fat deposition (Chen et al., 2017).

Growth rates have dramatically increased by over four times between 1957 and 2005 (Zuidhof et al., 2014) and modern chickens reach the market age in less than half the time that would have taken 60 years ago. This change is associated with hyperphagia and excessive fat deposition mainly in broiler breeders that require feed restriction and feed regimens (de Jong et al., 2002). In broiler breeders, obesity compromises reproductive functions and sexual activity, and thereby alters performance and welfare (De Jong and Guémené, 2011). On the other hand, feed restriction causes chronic hunger, stress, frustration, aggressiveness, cannibalism, and boredom (Decuypere et al., 2010). In commercial broilers, fat deposition reduces meat yield, feed efficiency, and alters meat quality, which all together results in profit margin loss (Lippens et al., 2000).

Generally fat deposition originates from exogenous (diet) and/or endogenous (adipogenesis/lipogenesis) sources. As commercial broilers are fed lipid-poor diets (<10%), the majority of the accumulated fat is derived from the liver, which is the main site for *de novo* lipogenesis (Goodridge and Ball, 1967; Leveille et al., 1968; Yeh and Leveille, 1971). Fat metabolism is modulated through a tight interaction between synthesis and degradation programs, which both are controlled by exogenous factors (diet, environment, etc.,) and endogenous complex molecular mechanisms and pathways that are not fully defined in avian species. In the Research Topic, we invited experts in their fields and gathered outstanding and elegant reviews, case reports, and breakthrough research articles to provide new insights into fat metabolism of various avian species (chickens, quails, ducks) at peripheral and central levels from different, yet complementary disciplines (physiology, genetics, and nutrition).

At the central level, Kato et al. showed that two-week intracerebroventricular infusion of neurosecretory protein GM (NPGM) increased body mass, and the mass of abdominal and gizzard fat in chickens, without effects on feed intake. At molecular levels, they showed that NPGM might induce hepatic fat deposition via down regulation of the hepatic PPARα gene expression.

At the peripheral level, the Bonnefont group has used a high throughput metabolomics approach to identify hepatic biomarkers for Foie Gras quality in duck. A total of eighteen quality-associated metabolite signatures were determined, with five specific to liver weight and four specific for technological yield at cooking. Massimino et al., on the other hand, used high throughput real-time quantitative PCR to determine the effect of embryonic thermal manipulation on the hepatic lipid and carbohydrate metabolism, stress, cell proliferation, and thyroid hormone pathways in mule ducks. They identified several key genes that might be involved in thermal long-term programming of hepatic metabolism. Surugihalli et al. used mass spectrometry-based metabolomics to evaluate the rapid remodeling of hepatic mitochondrial and cytoplasmic networks in chicken E18 embryo and d3 post-hatch chicks. Several metabolites were profiled in both plasma and liver showing a transition from lipid oxidation in embryonic liver to *de novo* lipogenesis in neonatal liver. Using forced molting laying hens, cecal metabolomics, and liver transcriptomics, Ruirui (abstract only) identified regulatory intestinal-liver lipid metabolism factors affecting reproductive performance in laying hens.

In adipose tissue, Zhao et al. determined the developmental changes of adipocyte differentiation, lipid synthesis, lipolysis, fatty acid β-oxidation, and lipid content from chicken embryo day 12 to day-9 post hatch. They showed that the mitochondrial copy number and fatty acid β-oxidation increased during the post hatch period, indicating that subcutaneous adipose tissue plays an important role in energy supply. Sun et al. used the Iso-Seq technology and identified several long non-coding RNA and alternative splicing in abdominal and subcutaneous adipose tissues of pekin ducks. Na et al. reported several reference genes for real-time quantitative PCR in chicken adipose tissue and adipocytes. Kim et al. determined the effect of *in ovo* injection of different doses of all-trans retinoic acid on quail embryonic adipogenesis. They found that all-trans retinoic acid promoted hypertrophic fat accretion in quail embryos via upregulation of PPARγ and FABP4 and down regulation of Dlk1. The Gilbert group studied the effect of dietary baicalein supplementation on adipose tissue gene expression profiles during the first week post hatch in chickens. They showed a reduction of growth performance (body weight, feed intake), breast muscle weight, and subcutaneous and abdominal fat weights along with a modulation of several genes involved in adipogenesis and fat storage. In a separate paper, the Cline group reviewed the effects of dietary flavonoids on lipid metabolism in liver, skeletal muscle, and adipose tissue of poultry species. Finally, Kim and Voy provided a thorough review associated with the beneficial effects of n-3 polyunsaturated fatty acids in reducing fat accretion in poultry.

In summary, the papers within the current Research Topic provide new insights and discoveries related to lipid metabolism in various avian species (chicken, quail, and duck) and suggest some solutions and perspectives for future investigations aiming to reduce fat accretion in poultry. It is my fervent hope that this ebook and the Research Topic is a great resource for the readers, and we look forward to a second volume to expand more research and knowledge associated with other molecular pathways of lipid metabolism in various other avian species.
